# Thermal and solutal heat transport investigations of second order fluid with the application of Cattaneo-Christov theory

**DOI:** 10.1371/journal.pone.0304794

**Published:** 2024-07-11

**Authors:** Hossam A. Nabwey, Aamir Abbas Khan, Muhammad Ashraf, A. M. Rashad, Zeinab M. Abdelrahman, Miad Abu Hawsah

**Affiliations:** 1 Department of Mathematics, College of Science and Humanities in Al-Kharj, Prince Sattam bin Abdulaziz University, Al-Kharj, Saudi Arabia; 2 Department of Basic Engineering Science, Faculty of Engineering, Menoufia University, Shebin El-Kom, Egypt; 3 Department of Mathematics, University of Sargodha, Sargodha, Pakistan; 4 Department of Mathematics, Aswan University, Faculty of Science, Aswan, Egypt; 5 Basic and Applied Sciences Department, College of Engineering and Technology, Arab Academy for Science & Technology and Maritime Transport (AASTMT), Aswan Branch, Aswan, Egypt; Beijing University of Technology, CHINA

## Abstract

The present examination of mass and heat communication looks at the impact of induced magnetic field, variable thermal conductivity, and activation energy on the flow of second-order liquid across a stretched surface. The mass-heat transfer is also treated using the Model for generalized Fourier and Fick’s Laws. The model equations are transformed as needed to produce a system of nonlinear ODEs, which are then numerically solved with the help of BVP4C integrated MATLAB approach. The heat-mass flow parameters are analyzed by the table and graphs. An increment in the estimations of 2^nd^ grade fluid parameter (*β*) with magnetic field parameter (*M*) increase the speed sketch. For the stronger estimations of Schmidt number (*S*_*c*_), parameter of magnetic field (*M*) and Eckert number (*E*_*c*_) have the growing behavior on the temperature profile.

## 1. Introduction

The non-Newtonian fluid in such substances that do not fulfill Newtonian’s law of viscosity. Aspect and behavior of the non-Newtonian fluids continue to snatch extensive appreciation owing to its vast consequences in engineering and manufacturing purposes. Particularly, such type of fluids is experienced in recommended fiber technology, chemical and nuclear industries, shampoos, material processing, physiology, pharmaceuticals, oil reservoir engineering, foodstuffs, pharmaceuticals and nuclear industries etc. Instances of such specific fluids are polymer solutions, oil reservoirs, crystal growth, grease, coating of wires, paints, blood a lower shear rate, grease, coating of wires, milk, and lot of others. The features of non-Newtonian liquids cannot be effectively explained by the well-established Navier-Stokes theory. For the purpose of better understand the characteristics of non-Newtonian liquids, several models have been created. Three categories, best-known as the rate, differential, and rate types, have been established for these non-Newtonian fluid models. Differential fluids are the most straightforward class of non-Newtonian fluids. The 2^nd^-grade fluid model from the differential type of non-Newtonian liquids is a part of the current work. Shi-junLio [1] discussed the non-Newtonian fluid flows brought on by a stretched sheet with the consequence of a magnetic field while also developing the analytical solution utilizing homotopy analysis. The erratic flow of non-Newtonian fluid with the influence of the magnetic field was described by Xu and Liao [2] using the analogy of an impulsively stretched sheet. Xu and Liao [3] investigated the heat-mass communication of non-Newtonian fluid flow in response to the stretched sheet. Sahoo [4] explored the heat-mass transfer into a 3^rd^-grade fluid (non-Newtonian) stagnation point flow with partial slip effect brought on by a linearly stretched sheet using the shooting and Broyde procedures. By means of an extended sheet, Javed et al. [5] looked at the flow of a Powell and Eyringfluid flow. The properties of two-dimensional steady Jeffery nanofluid flows close to a vertically elongating surface were studied by Zhang et al. [6]. Taking into account the effective thermal effects of a non-uniform internal heat source, they concentrated on situations where thermal radiation has a linear impact on the viscoelastic nanofluid flows driven by the surface. El Harfouf et al. [7] conducted a computational study on the flow of a squeezing nanofluid. They examined how factors such as thermal radiation, magnetohydrodynamics (MHD), and chemical processes influence the flow within a constrained parallel-wall geometry. Reddy et al. [8] conducted an analysis of the mass-heat transportation into a Cassonfluid flow with the consequences of a stretched surface, thermal diffusion, and magnetic field. Ashraf et al. [9] conducted a theoretical study on the lifting and drainage of a third-grade fluid that includes surface tension. Unsteady radiative magnetohydrodynamic flow of a non-Newtonian Casson hybrid nanofluid across a vertically moving porous surface with exponential acceleration was studied by Krishna et al. [10] and Wakif et al. [11]. They considered slip velocity in a revolving frame. Rashidi et al. [12] scrutinized the formation of entropy across a stretched sheet while observing the convective flow of a 3^rd^-order liquid with magnetic field influence. Some other related lately research articles to non-Newtonian fluids are expressed (See Refs. [12–16]).

Investigation of boundary layer movement through joining mass and heat transportation past a stretching sheet has acquired great attention by several engineers and scientists in the modern years for the reason that its various significant practical investigators in many manufacturing processes. Such applications take account of wire glass blowing, distillation of towers, cooling of electronic chips, exclusion of plastic sheets, aerodynamic, glass blowing, and artificial fibers etc. Design of chemical processing equipment, crop damage from freezing, fog formation and dispersion, moisture and temperature distribution over agricultural arenas and orchards of fruit trees, chilling tower construction, and food processing all heavily rely on the interaction of chemical reaction and thermophoresis with mass and heat transport. The cooling towers are a cost-effective technique to manage enormous amounts of water. When the gap between surface temperature and temperature increases, the influence of the polymer processing industry on controlling the heat transportation process in thermal radiation increases. In light of the effects of heat generation or absorption as well as chemical reactions brought on by the extended surface, Liu [17] explored the mass-heat transmission of the hydromagnetic flow. According to Khan et al. [18], the mass-heat transfer in the 2^nd^-grade fluid flow was studied in relation to a uniform magnetic effect, a heat sink / source that is temperature dependent, viscous dissipation, and a permeable medium because of a porous stretched sheet. Afify [19] investigated how the ensuing heat and mass transport was influenced by a continuous magnetic field, chemical reaction, and natural and free convectional flow of a incompressible, viscous, and electrically conducting fluid. Sanyayanand and Khan [20] addressed the mass-heat communication into a viscoelastic fluid flow using an exponentially expanding sheet. A unique theoretical model for tri-hybrid nanofluids was presented by Samad, and Mohebujjaman [21] in an effort to enhance heat transmission. Samad and Mohebjjaman [22] looked at the specifics of how mass and heat are transferred into a steady, 2D, free convectional flow of an incompressible and viscous fluid in a high-powered state of a buoyancy force, a uniform magnetic effect, and heat generation by an isothermal linearly extending sheet. In order to account for heat radiation in an unstable magnetohydrodynamic (MHD) boundary layer scenario, Madhu and colleagues [23] studied the flow of a non-Newtonian Maxwell nanofluid over a stretched surface. The mass and heat transmission have been done by some researchers for different physical dominating parameters on the stretching sheet in the study [24–29].

A magnetic field can occasionally be produced by moving fluids, however because metals and partly ionised fluids have very low magnetic Reynolds numbers, this effect is often quite weak. But this generated magnetic field becomes significant and must be taken into account when the magnetic Reynolds number is one or higher. In several fields, including engineering, astrophysics, and geophysics, it is extremely important. It influences, for instance, the flow of fluids within the Earth, the formation of stars, the behavior of spinning magnetic stars, and even uses such as the containment of plasma in fusion reactors and the comprehension of phenomena like planetary motions and solar dynamos. We are concentrating on fluids with high magnetic Reynolds numbers for our investigation because of the substantial influence of generated magnetic fields in these regions. The movement of a fluid with certain viscosity properties and the ability to transmit electricity through a porous material along a vertical stretching surface were studied by Krishna et al. [30]. They examined the effects of thermophoresis, thermal radiation, and circumstances at the fluid-fluid border where heat enters and exits the system on this flow. The influence of Hall and ion slip effects on the motion of nanofluids in a porous media past a revolving, vertical flat plate was investigated by Krishna and Chamkha [31]. Solar dynamo, magnetohydrodynamics power generation, plasma confinement, crude oil purification, and spinning magnetic stars are only a few of the scientific and technical difficulties that Kumar et al. [32] investigated. They looked at magnetohydrodynamic mixed convection, thermal radiation, and viscous dissipation while studying the continuous flow of an incompressible, viscous, conducting fluid across a vertical plate. A thorough study of the Soret and Joule effects of magnetohydrodynamic mixed convective flow was carried out by Krishna et al. [33]. They examined, taking into account Hall effects, the flow of an incompressible, electrically conducting viscous fluid across an endless vertical porous plate. The behaviour of titanium dioxide-particle-containing ethylene glycol-based nanofluids flowing steadily close to a vertical permeable surface was studied by Wakif et al. [34]. They examined the behaviour of these fluids under both nonuniform internal heating and uniform blowing. A powerful technique known as the generalized differential quadrature local linearization approach was developed by Alghamdi et al. [35]. With the aid of this technique, they are able to analyze the mass and heat properties of electrically conducting nanofluids in a realistic way as they approach a vertically heated surface that is being impacted by an active electromagnetic actuator in a non-Darcian laminar fashion. Some more efforts regarding induced magnetic field effect for the flow of various fluids can be seen by Refs. [36–38].

The least amount of energy that may cause a particle to undergo a variety of changes or chemical reactions is referred to as activation energy. The activation energy is frequently characterized by the sign *E*_*a*_ and measured in kcal/mol or kj/mol. In food preparation, geothermal technology, oil reservoir, chemical engineering, and mechano chemistry, the measurement of activation energy is usually helpful. It is the smallest quantity of energy a volatile type species must keep in order enduring an explicit reaction and is a very worthwhile concept in petrochemical engineering processes, geothermal and chemical. Awad et al. [39] analyzed an investigation of the mass-heat transmission into the rotating, incompressible, viscous fluid flow with binary chemicaland reactionactivation energy by a stretched sheet. The influence of activation energy and velocity slip on the 3DErying-Powell fluid flow were described using the stretching sheet approach by Umar et al. [40]. By using a stretching sheet and a Catteno-Christov double diffusion thermophoretic, Ali et al. [41] offered evidence for the consequence of activation energy on the transient rotational flow of Maxwell fluid with nanoparticles under the influence of Brownian motion. Zaib et al. [42] studied the flow of a generalized Newtonian Carreau liquid using nonlinear thermal radiation, nanoparticles with binary chemical processes, and activation energy, towards a nonlinear stretched surface. In their inquiry of the Jaffery nanofluid flow, Hayat et al. [43] discussed how entropy is generated by stretching sheets of various thicknesses under the influence of joule heating, brownian motion, viscous dissipation, thermophoresis, and activation energy. Some more efforts regarding activation energy effect for the flow of various fluids can be seen by Refs. [44–48].

Examining the consequences of an induced magnetic field influence on a second-order fluid flow when activation energy, Catteno-Christov, and fluctuating thermal conductivity are in play is the objective of the current inquiry. Additionally taken into account are the boundary conditions for velocity and thermal slippage. Subsequently, converting the couple PDEs into the system of non-linear ODEs through the appropriate transformations, the resulting system is computed by using a Matlab BVP4C technique. To investigate the relevance of the several dominant physical characteristics, graphs are created. From the literature overview, it is revealed that no such exploration is considered for yet.

## 2. Problem description

**Fig 1:**
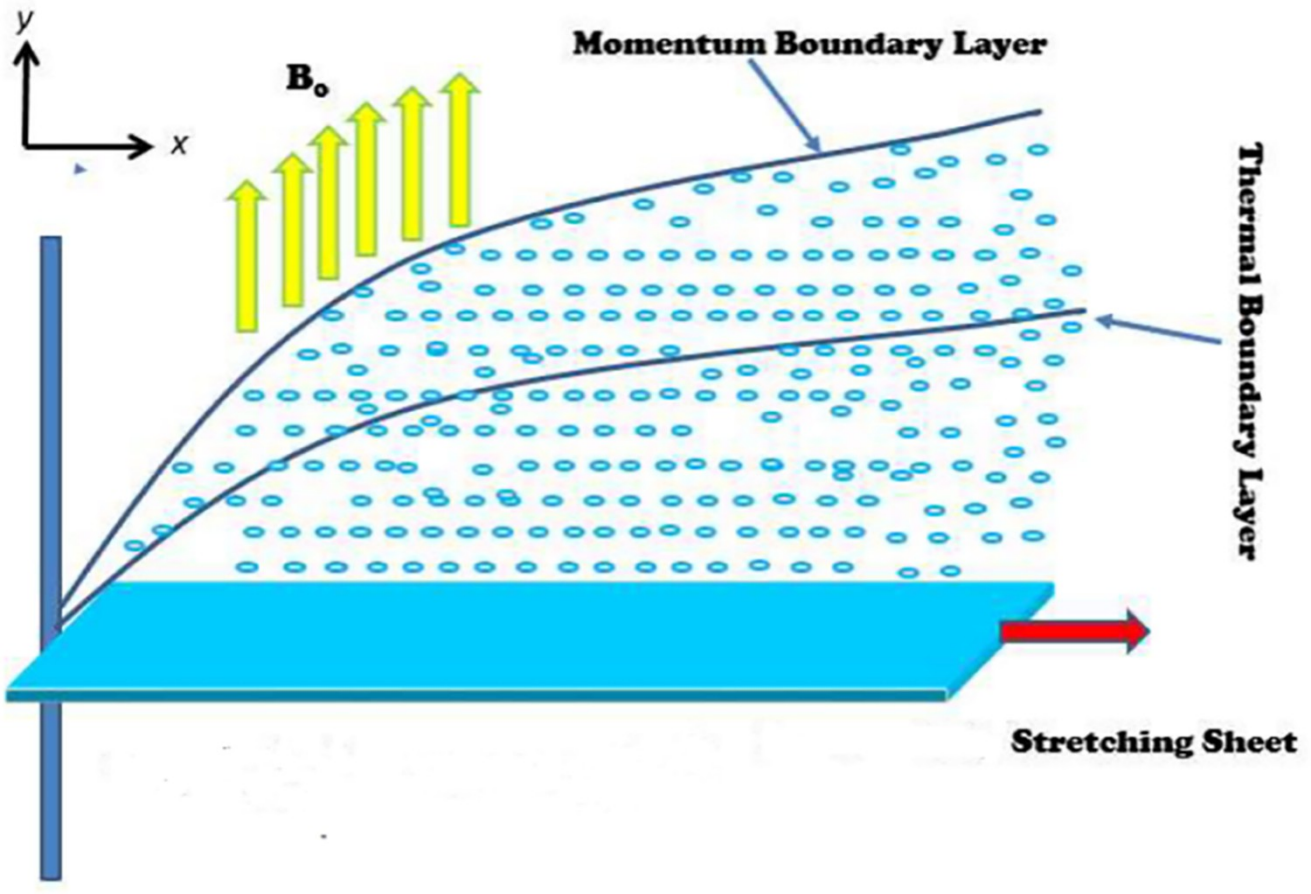
Flow mechanism.

The current inquiry aims to investigate the influence of activation energy, Catteno-Christov, and variable thermal conductivity on the flow of a second-grade fluid when an induced magnetic field is present. Additionally considered are the boundary conditions for thermal slip and velocity slip. The intensity of the magnetic field that is being used is constant *H*_0_ and the induced magnetic effect is used in y-direction and its parallel component is *H*_1_ and *H*_2_ is of its normal component (see in Fig 1).

The following are the governing boundary layer equations under the aforementioned presumptions: [49–51]

$$\frac{\partial u}{\partial x}+\frac{\partial v}{\partial y}=0,$$ (1)

$$\frac{\partial H_{1}}{\partial x}=-\frac{\partial H_{2}}{\partial y},$$ (2)

$$u\frac{\partial u}{\partial x}+v\frac{\partial u}{\partial y}=\nu \frac{\partial^{2}u}{\partial y^{2}}+\frac{\alpha_{1}}{\rho }\left[\frac{\partial }{\partial x}(u\frac{\partial^{2}u}{\partial y^{2}})+\frac{\partial u}{\partial y}\frac{\partial^{2}u}{\partial x\partial y}+v\frac{\partial^{3}u}{\partial y^{3}}\right]+\frac{\mu_{0}}{4\pi \rho_{f}}\left[H_{1}\frac{\partial H_{1}}{\partial x}+H_{2}\frac{\partial H_{1}}{\partial y}\right],$$ (3)

$$u\frac{\partial H_{1}}{\partial x}+v\frac{\partial H_{1}}{\partial y}=\alpha_{2}\frac{\partial^{2}H_{1}}{\partial y^{2}}+H_{1}\frac{\partial u}{\partial x}+H_{2}\frac{\partial u}{\partial y},$$ (4)

$$u(\frac{\partial T}{\partial x})+v(\frac{\partial T}{\partial y})+\lambda_{E}\left[\frac{\partial T}{\partial x}(v\frac{\partial u}{\partial y}+u\frac{\partial u}{\partial x})+\frac{\partial T}{\partial y}(u\frac{\partial v}{\partial y}+v\frac{\partial v}{\partial y})+2uv\frac{\partial^{2}T}{\partial x\partial y}+u^{2}\frac{\partial^{2}T}{\partial x^{2}}+v^{2}\frac{\partial v}{\partial x}\right]=\frac{1}{\rho C_{p}}\frac{\partial }{\partial y}\left[k(T)\frac{\partial T}{\partial y}\right]+\left[D_{B}\frac{\partial C}{\partial y}\frac{\partial T}{\partial y}+{\left(\frac{\partial T}{\partial y}\right)}^{2}\frac{D_{T}}{T_{\infty }}\right]+\frac{\mu }{\rho C_{p}}{(\frac{\partial u}{\partial y})}^{2}+\frac{\alpha_{1}}{\rho C_{p}}\frac{\partial u}{\partial y}\left[\frac{\partial }{\partial y}(v\frac{\partial u}{\partial y}+u\frac{\partial u}{\partial x})\right],$$ (5)

$$u\frac{\partial C}{\partial x}+v\frac{\partial C}{\partial y}+\lambda_{c}\left[(u\frac{\partial u}{\partial x}+v\frac{\partial u}{\partial y})\frac{\partial C}{\partial x}+(u\frac{\partial v}{\partial y}+v\frac{\partial v}{\partial y})\frac{\partial C}{\partial y}+2uv\frac{\partial^{2}C}{\partial x\partial y}+u^{2}\frac{\partial^{2}C}{\partial x^{2}}+v^{2}\frac{\partial^{2}C}{\partial y^{2}}\right]=D_{B}\frac{\partial^{2}C}{\partial y^{2}}+\frac{D_{T}}{T_{\infty }}(\frac{\partial^{2}T}{\partial y^{2}})-k_{1}^{**}(C-C_{\infty })-{K_{r}}^{2}{\left(\frac{T}{T_{\infty }}\right)}^{m}exp((\frac{-E_{a}}{kT}))(C-C_{\infty }),$$ (6)

Where, *k*(*T*) is the variable thermal conductivity is described as:

$$k(T)=k_{\infty }(1+\varepsilon \frac{T-T_{\infty }}{\Delta T}),$$ (7)

Boundary conditions

$$u=u_{w}+\lambda_{1}\frac{\partial u}{\partial y},v=0,H_{1}=0,\frac{\partial H_{1}}{\partial y}=H_{2},T=T_{w}+\lambda_{2}\frac{\partial T}{\partial y},C=C_{w}+\lambda_{3}\frac{\partial C}{\partial y},aty=0$$ (8)

$$u\to 0,T\to T_{\infty },C\to C_{\infty },H_{1}\to H_{\infty }\;\mathrm{at}\;y\to \infty$$

Similarly transformations

$$u=cxf^{\prime },v=-\sqrt{c\nu }f,H_{1}=H_{0}xg^{\prime },H_{2}=-\sqrt{c\nu }g,\theta =\frac{T-T_{\infty }}{T_{w}-T_{\infty }},\phi =\frac{C-C_{\infty }}{C_{w}-C_{\infty }},\eta =\sqrt{\frac{a}{\nu }}y$$ (9)

Where, $(x,y),(u,v),(H_{1},H_{2}),\nu ,\alpha_{1},\rho ,\mu_{0},\alpha_{2},T,C_{p},k(T),D_{B},C,\lambda_{H},\lambda_{m},k_{1}^{**},\mu ,q_{r},C_{\infty },k_{r}^{2},m,E_{a},K,\lambda_{1},\lambda_{2},$ and *λ*_3_ are Cartesian coordinates, components of speed, components of the induced magnetic field between the x- y-axes, kinematic viscosity, second grade fluid coefficient, density of the fluid, magnetic permeability, magnetic diffusivity, temperature of the liquid, thermal conductivity, Brownian motion factor, concentration of fluid, thermophoresis coefficient, ambient temperature, dynamic viscosity, the coefficient of thermal radiation, the moment when the heat flow relaxes, the time of relaxation of mass flux, chemical reaction coefficient, ambient concentration, chemical reaction rate constant, fitted rate constant, activation energy coefficient, reaction rate constant, coefficient of velocity slip, parameter of thermal slip and concentration slip parameter respectively.

Through the similarity transformations of Eq (9) in the Eqs (3)–(6), are reduced as followed as,

$$f^{\prime \prime \prime }-2{f^{\prime }}^{2}+ff^{\prime \prime }+\beta (3f^{\prime }f^{\prime \prime \prime }+\eta f^{\prime \prime }f^{\prime \prime \prime }-\frac{1}{2}ff^{\left(iv\right)})+M(2{g^{\prime }}^{2}-gg^{\prime \prime })=0$$ (10)

$$\lambda g^{\prime \prime \prime }+g^{\prime \prime }f-f^{\prime }g=0$$ (11)

$$(1+\epsilon \theta )\theta^{\prime \prime }+\epsilon {\theta \prime }^{2}-P_{r}(f^{\prime }\theta -f\theta^{\prime })-\delta_{e}P_{r}(ff^{\prime }\theta^{\prime }+f^{\prime }\theta^{\prime \prime })+P_{r}(N_{b}\theta^{\prime }\phi^{\prime }+N_{t}{\theta^{\prime }}^{2})+P_{r}E_{c}[{f^{\prime \prime }}^{2}+M{f^{\prime }}^{2}+\beta f^{\prime \prime }(f^{\prime }f^{\prime \prime }-ff^{\prime \prime })]+(1+\frac{4}{3}R)\theta^{\prime \prime }=0$$ (12)

$$\phi^{\prime \prime }+\frac{N_{t}}{N_{b}}\theta^{\prime \prime }-f^{\prime }\phi +f\phi^{\prime }+S_{c}\delta_{c}(ff^{\prime }\phi^{\prime }+f^{2}\phi^{\prime \prime })-S_{c}K_{r}\phi -S_{c}\Omega \phi {\left(1+\Gamma \theta \right)}^{n}exp(\frac{-A_{e}}{1+\Gamma \theta })=0$$ (13)

Corresponding boundary conditions are as followed,

$$\begin{array}{l} f^{\prime }=1+S_{1}f^{\prime \prime },f=0,g=0,\theta =1+S_{2}\theta^{\prime },\;g^{\prime \prime }=0,\;\phi =1+S_{3}\phi^{\prime }\;\mathrm{at}\;\eta \to 0\\ f^{\prime }=0,\theta =0,\phi =0,g^{\prime }=1,g=0\;at\;\eta \to \infty . \end{array}$$ (14)

$\beta =\frac{\alpha_{1}a}{\rho \upsilon }$ (Second grade fluid parameter), $M=\frac{\mu_{0}H_{0}l}{4\pi \rho }\sqrt{\frac{\sigma }{\mu_{0}}}$ (Magnetic parameter), $\lambda =\frac{1}{4\pi \sigma \mu_{0}\upsilon }$ (Magnetic prandtl number), *ε* = *γ*(*T*_*w*_−*T*_*∞*_) (Variable thermal conductivity), $P_{r}=\frac{\mu c_{P}}{k}$ (Prandtl Number), *δ*_*e*_ = *aλE* (Parameter of time relaxation of heat flux), $N_{b}=\frac{D_{B}(C_{w}-C_{\infty })}{\nu }$ (Brownian motion parameter), $N_{t}=\frac{D_{T}(T_{w}-T_{\infty })}{\nu T_{\infty }}$ (Thermophoresis parameter), $E_{c}=\frac{a^{2}x^{2}}{c_{P}(T_{w}-T_{\infty })}$ (Eckert number), $R=\frac{4\sigma^{*}T_{\infty }^{3}}{kk^{*}}$ (Parameter of thermal radiation), $S_{c}=\frac{\nu }{D_{B}}$ (Schmidt number), $\Omega =\frac{k_{r}^{2}}{a}$ (Reaction rate constant), $S_{1}=\lambda_{1}\sqrt{\frac{a}{\nu }}$ (parameter of velocity slip), $S_{2}=\lambda_{2}\sqrt{\frac{a}{\nu }}$ (parameter of thermal slip), $S_{3}=\lambda_{3}\sqrt{\frac{a}{\nu }}$ (parameter of concentration slip).

### 2.1. Physical quantities

The physical quantities are absolutely necessary in engineering. The Skin Friction, Nusselt, and Sherwood numbers are as follows:

$$C_{fx}=\frac{\tau_{w}}{\frac{1}{2}\rho {u_{w}}^{2}},$$ (15)

$$\tau_{w}={\left[\left(\frac{\partial u}{\partial y})\mu +\rho \alpha_{1}(\frac{\partial^{2}u}{\partial x\partial y}u+2\frac{\partial u}{\partial y}\frac{\partial u}{\partial x}\right)\right]}_{|y=0},$$ (16)

$${C_{fx}}_{|\eta =0}={\left(\frac{R_{e}}{2}\right)}^{-\frac{1}{2}}[1+3\alpha f^{\prime }(0)]f^{\prime \prime }(0),$$ (17)

$$N_{ux}=\frac{xq_{w}}{k(T-T_{\infty })},$$ (18)

$$q_{w}={\left|-k(\frac{\partial T}{\partial y})\right|}_{y=0},$$ (19)

$$N_{ux}=-\sqrt{\frac{x}{l}}{\left(\frac{R_{ex}}{2}\right)}^{-\frac{1}{2}}\theta^{\prime }(0),$$ (20)

$$S_{hx}={\left|-\frac{x}{\left(C_{w}{-C}_{\infty }\right)}(\frac{\partial C}{\partial y})\right|}_{y=0}.$$ (21)

$$S_{hx}=-\sqrt{\frac{x}{l}}{\left(\frac{R_{ex}}{2}\right)}^{-\frac{1}{2}}\phi^{\prime }(0).$$ (22)

Where, $R_{ex}=\frac{{xu}_{w}}{\nu }$ is the Renolds number.

## 3. Numerical scheme

**Fig 2:**
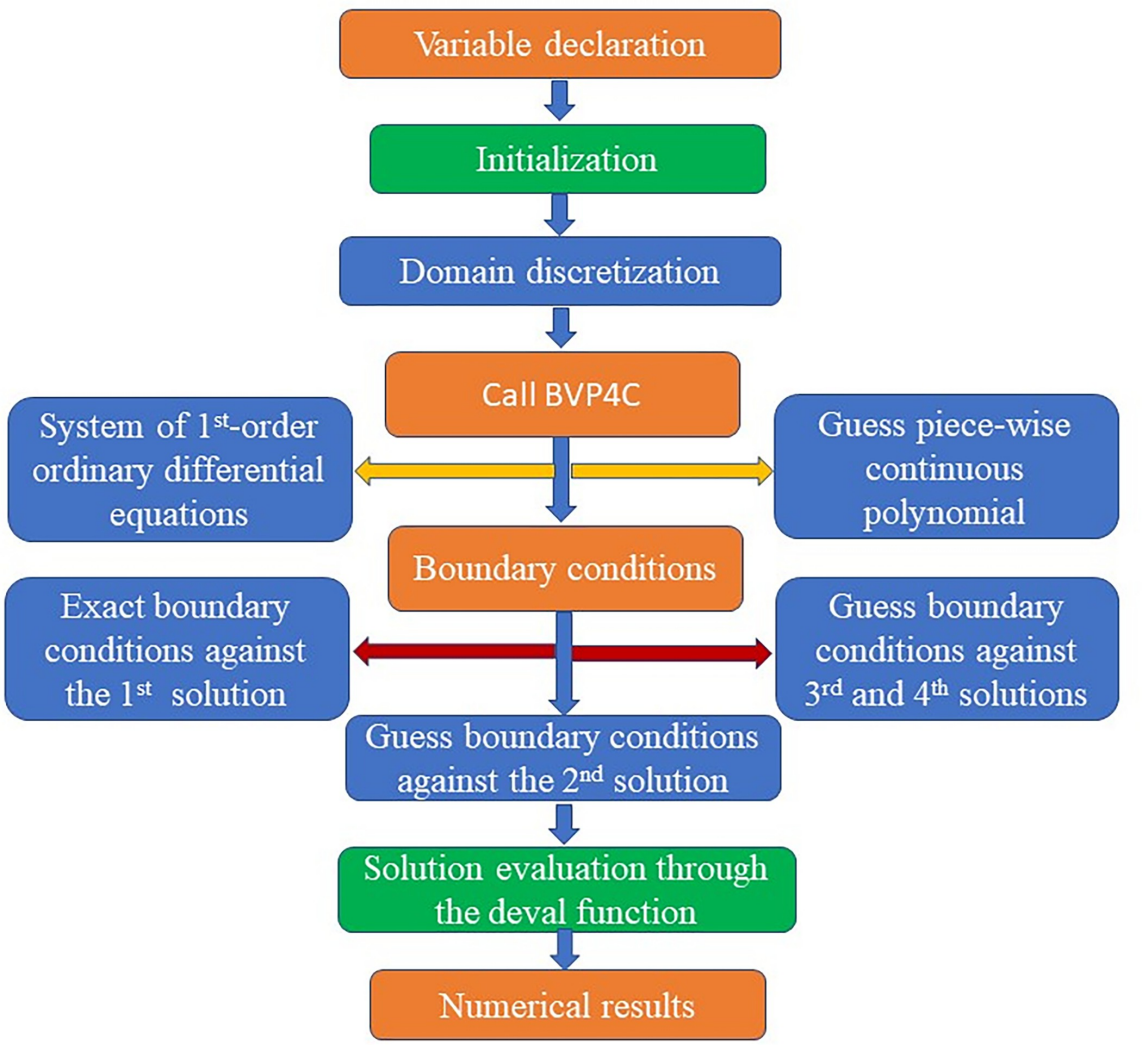
Numerical scheme.

The Numerical Scheme is as shown in Fig 2.

## 4. Method of solution

It is advised to use this section to solve well-known ODEs for flow, temperature, concentration, and concentration of microorganisms numerically. A built in MATLAB function BVP4C is applied for these solutions. Upon careful consideration, a step size of 0.001 and convergence conditions of 10^−6^ were selected. This function requires the first order ODEs to workout thus, to convert a provided, the following variables are used equation into 1^st^ order:

$$f=y_{1},f^{\prime }=y_{2},f^{\prime \prime }=y_{3},f^{\prime \prime \prime }=y_{4},f^{\left(iv\right)}={yy}_{1},$$ (23)

$$g=y_{5},g^{\prime }=y_{6},g^{\prime \prime }=y_{7},g^{\prime \prime \prime }={yy}_{2},$$

$$\theta =y_{8},\theta^{\prime }=y_{9},\theta^{\prime \prime }={yy}_{3},$$

$$\phi =y_{10},\phi^{\prime }=y_{11},\phi^{\prime \prime }={yy}_{4},$$

The system of first order ODEs is: (23)

$${yy}_{1}=\frac{2}{\beta y_{1}}\{y_{4}-2y_{2}^{2}+y_{1}y_{3}+\beta (3y_{2}y_{4}+\eta y_{3}y_{4})+M(2y_{6}^{2}-y_{5}y_{7})\},$$ (24)

$${yy}_{2}=\frac{1}{\lambda }\{y_{2}y_{5}-y_{7}y_{(25)1}\},$$ (25)

$${yy}_{3}=\left[\frac{1}{\left(1+\epsilon y_{8})-\delta_{e}P_{r}y_{2}+(1+\frac{4}{3}R\right)}\right]\{P_{r}(y_{2}y_{8}-y_{1}y_{9})-\epsilon y_{9}^{2}+\delta_{e}P_{r}y_{1}y_{2}y_{9}-P_{r}(N_{b}y_{11}y_{9}+N_{t}y_{9}^{2})-P_{r}E_{c}(y_{3}^{2}+My_{2}^{2}+\beta y_{3}(y_{2}y_{3}-y_{1}y_{3}))\},$$ (26)

$${yy}_{4}=\frac{1}{\left(1+\delta_{e}S_{c}y_{1}^{2}\right)}\left\{y_{2}y_{10}-\frac{N_{t}}{N_{b}}{yy}_{3}-y_{1}y_{11}-\delta_{e}S_{c}y_{1}y_{2}y_{11}+k_{r}S_{c}y_{10}+S_{c}\Omega {\left(1+\Gamma y_{8}\right)}^{n}e^{-(\frac{A_{e}}{1+\Gamma y_{8}})}\right\}.$$ (27)

$$y_{1}(0)=S_{1}+y_{2}(0),y_{1}(0)=0,\;y_{1}(0)=0,y_{5}(0)=0,$$ (28)

$$y_{8}(0)=1{+S}_{2}y_{9}(0),{y_{7}(0)=0,y}_{10}(0)=1{+S}_{3}y_{11}(0),$$

$$y_{2}(\infty )=0,\;y_{5}(\infty )=0,\;y_{6}(\infty )=1,\;y_{8}(\infty )=0,\;y_{10}(\infty )=0.$$

## 5. Result and discussion

**Fig 3:**
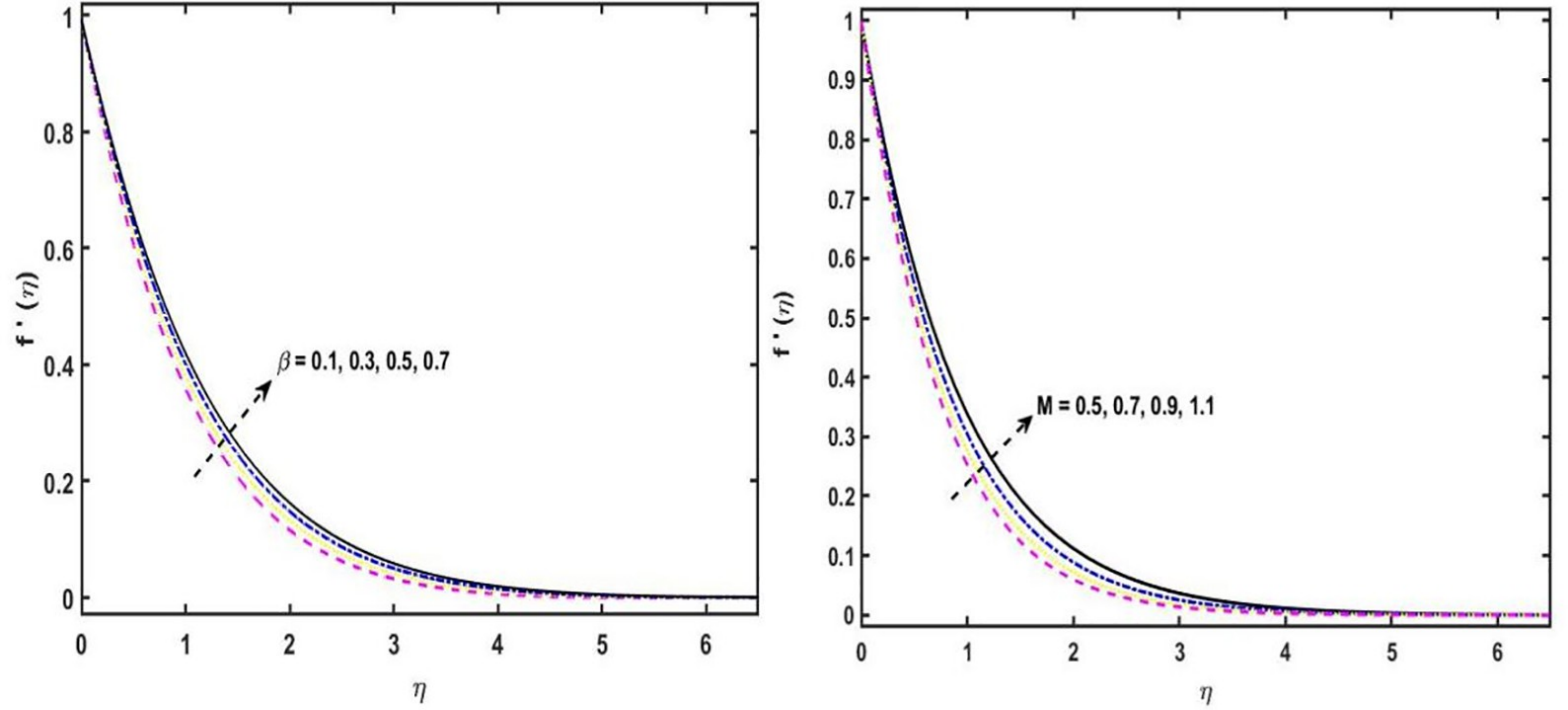
(a). f′(η) sketch for distinct variations of *β*. (b). f′(η) sketch for distinct variations of *M*.

**Fig 4:**
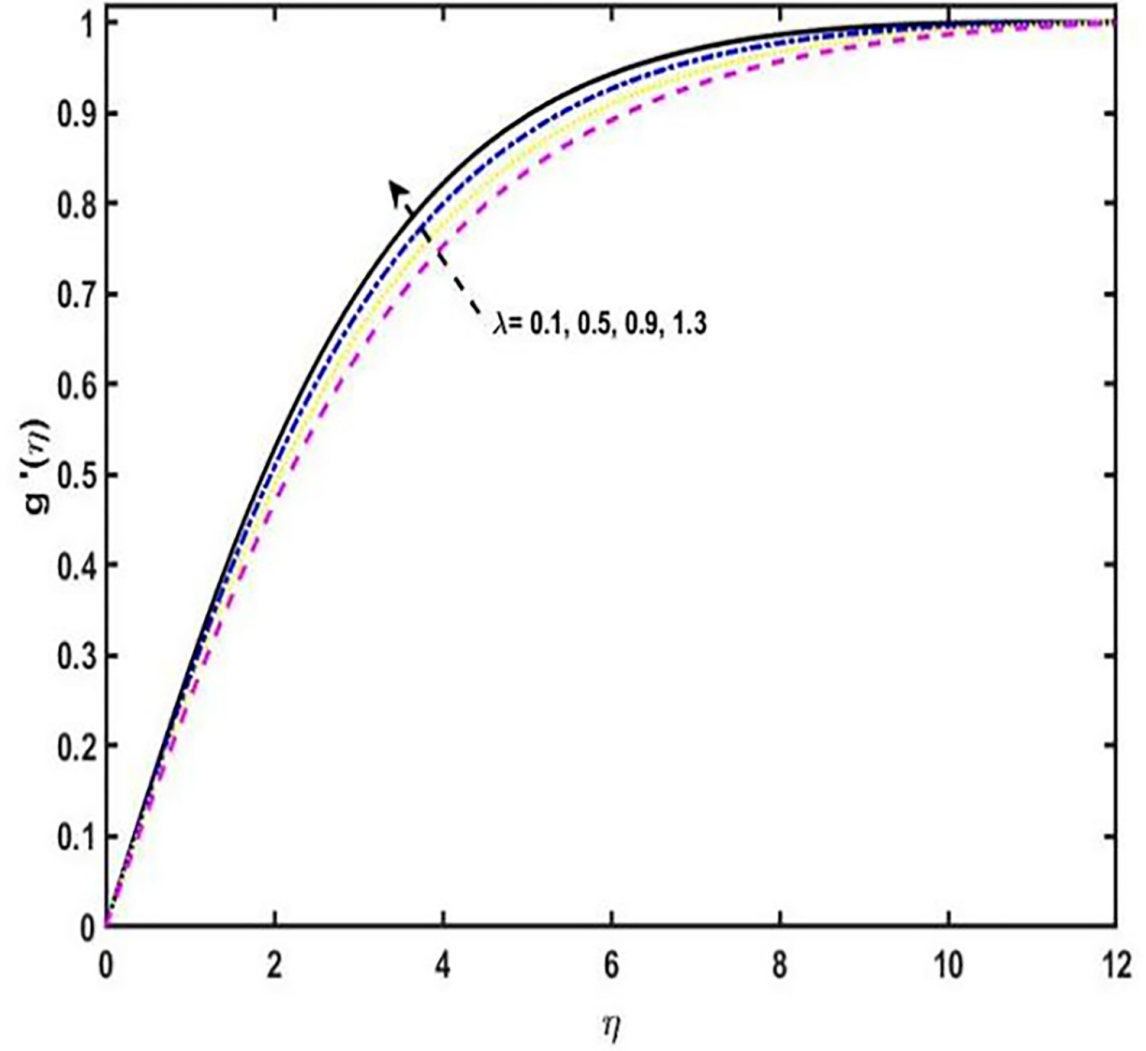
Induced magnetic field sketch for distinct variations of *λ*.

The present research segment exposes the graphical and tabulated data aspect of many related variables on the induced magnetic, temperature, concentration and velocity sketches. Fig 3(A) describes the velocity sketch for the diverse values of 2^nd^ order fluid parameter *β*. Physically, it has been distinguished that by growing the estimations of *β*, the velocity sketch boosts due to the viscosity of the momentum boundary layer width. The velocity sketch for the modification of the magnetic field parameter is shown in Fig 3B). The velocity profile is said to have risen consequently of the higher estimations of *M*. The magnetic field alienates the boundary layer, increasing its thickness and, as a result, the fluid velocity. For the various iterations of the reciprocal of the induced Prandtl number, the induced magnetic field is exhibited in Fig 4. The magnetic parameter has a positive physical effect on average skin-friction profiles, but the magnetic Prandtl number has the opposite effect.

**Fig 5:**
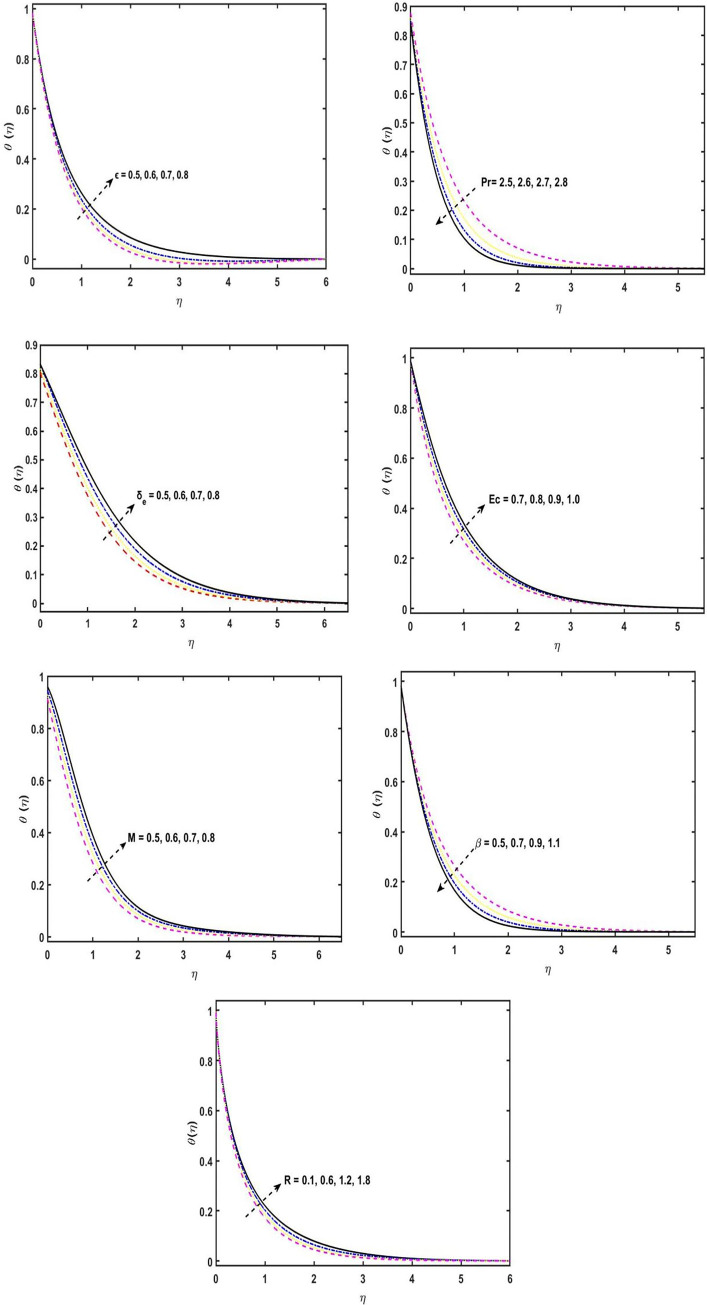
(a). Temperature sketch for distinct variations of *ε*. (b). Temperature sketch for distinct variations of *P*_*r*_. (c). θ(η) sketch for distinct variations of *δe*. (d). θ(η) sketch for distinct variations of *E*_*c*_. (e). θ(η) sketch for distinct variations of *M*. (f). θ(η) sketch for distinct variations of *β*. (g). θ(η) sketch for distinct variations of *R*.

When *λ* is augmented, it is perceived that the *g*′(*η*) profile grows. Fig 5(A) ravels the temperature sketch for the variations of the variable *ε*, as a result, an increase in the temperature sketch, by means of build up the wideness of the thermal boundary layer. The Fig 5(B) displays the temperature profile for the distinct estimations of the Prandtl number *P*_*r*_. It has been observed that as *P*_*r*_ varies more widely, the temperature sketch decreases. Physically, the Prandtl number is the ration between thermal diffusivity to the momentum diffusivity, Therefore, the temperature sketch diminishes by the stronger values of *P*_*r*_. By increasing in the variations of the *P*_*r*_, the outcome, the thermal boundary layer width diminishes. The consequences of the *δ*_*e*_ on the temperature sketch is seen in Fig 5(C). It is seen that, for the stronger estimations of *δ*_*e*_, the energy boundary layer and the temperature of the liquid decreases. Physically, for the higher estimations of the thermal relaxation parameter, the fluid particles require extra apparently to transfer of heat to its next to the particle. The Fig 5(D) exhibits the consequence of the Eckert number on the temperature distribution profile. Noted is the fact that, for the higher values of *E*_*C*_, the outcome, the temperature profile enhances. Actually, *E*_*C*_ number is the relation of the heat enthalpy differential and flow kinetic energy, therefore, enhancement in the Eckert number effects increment in the kinetic energy, and the average kinetic energy is used to define temperature. So, on the other hand, temperature of the fluid increases. It can be analyzed from the sketch that the temperature of the liquid rises if the estimations of the Eckert number upsurges. Fig 5(E) illustrates the upshot of the magnetic field parameter on the temperature profile. The temperature sketch is shown to grow for M estimations that are stronger. Physically, the magnitude of the velocity sketch in the boundary layer reduces by growing the estimations of the parameter of magnetic field, therefore, the temperature in the boundary layer would increase. The consequence of the parameter of the second grade fluid to the temperature sketch is shown in the Fig 5(F). It has been noted that by increasing the estimations of the second grade fluid parameter, the temperature profile reduces. Fig 5(G) represents that the significance of the thermal radiation parameter *R* on temperature profile. This is what is noticed, for the higher variations of the parameter of the thermal radiation *R*, due to this, the temperature field increases. Actually, the rate of heat transmission reduces by increasing the thermal radiation parameter.

**Fig 6:**
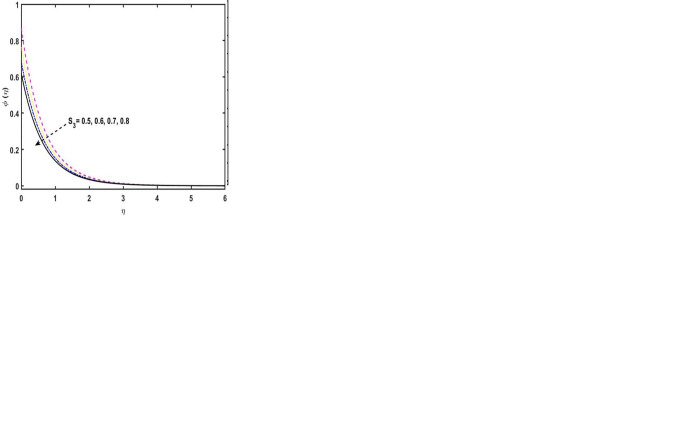
(a). Concentration sketch for distinct variations of *S*_*c*_. (b). Φ(η) sketch for distinct variations of *δ*_*c*_. (c). Concentration sketch for distinct variations of *A*_*e*_. (d). Velocity sketch for distinct variations of *S*_1_. (e). Temperature sketch for distinct variations of *S*_2_. (f). Concentration sketch for distinct variations of *S*_3_.

Fig 6(A). represents the consequences of the parameter of the Schmidt number on the concentration sketch. It is noted that, by enhancing the variations of the Schmidt number, as a result, the concentration sketch reduces. Given that the Schmidt number is the relationship of momentum to mass diffusivity. Thus, the concentration field boosts, for the greater estimations of the Schmidt number. Fig 6(B) demonstrations the consequence of *δ*_*c*_ on the concentration sketch. It is noticed that, for the higher estimations of the *δ*_*c*_, the concentration profile reduces. Physically, the increment in *δ*_*c*_ causes boost in retardation time and hence the concentration boundary layer thickness escalates. The consequence of the activation energy parameter to the concentration sketch is seen in the Fig 6(C). An interesting outcome is detected by increasing the variations of *A*_*e*_, due to this, the concentration profile increases. Actually, by virtue of upsurge in the parameter of the activation energy parameter guides to an growth the thermal reaction rate, because, the concentration profile boosts. The Fig 6(D) elucidates that the impression of velocity slip parameter on the velocity sketch. It is found that the velocity slip parameter decreases the velocity sketch. Fig 6(E) shows how the thermal slip parameter affects the temperature sketch. It is noted that for the higher estimations of the thermal slip parameter, because, the temperature sketch declines. Actually, by enhancing the thermal slip parameter cause diminishes in the heat-mass communication rates. The impact of the setting for the concentration slip on the concentration sketch is seen in Fig 6(F). The concentration profile is shown to drop when the concentration slip parameter is increased. Table 1 displays the behavior of skin friction for some diverse numerical estimation of some parameters. From the numerical values of second grade fluid parameter (*β*), the skin friction coefficient upsurges. Further, both magnetic field parameter (*M*) and reciprocal Prandtl number (*λ*) have the increasing behavior on the coefficient of skin friction and there is no impact of the Eckert number (*E*_*c*_) to the skin friction coefficient. Table 2 demonstrates the comparison estimations of skin friction against different variations of second grade fluid parameter (*β*), magnetic field parameter (*M*), radiation parameter (*R*), and thermal relaxation time parameter (*δ*_*e*_). The results presented in Table 2 align well with those reported in a previous study by Hossam et al. [52].

**Table 1:** Skin friction versus variations of distinct dominating parameters.

*β*	*M*	*λ*	*E*_*c*_	$C_{f_{x}}$
0.2s				2.38804
0.4				2.57353
0.6				2.74866
	1.1			1.36590
	1.2			1.35032
	1.3			2.33959
		0.3		2.35932
		0.4		2.34794
		0.5		2.32085
			1.5	2.35932
			2.0	2.35932
			2.5	2.35932

**Table 2:** Comparison estimations of Skin friction for distinct variations of *β*,*M*,*R* and *δ*_*e*_.

*β*	*M*	*R*	*δ*_*e*_	Present result [$C_{f_{x}}$]	Hossam et al. [52] [$C_{f_{x}}$]
0.1				1.7620	1.8230
0.2				1.8703	1.9071
0.3				1.9532	1.9900
	0.5			1.7572	1.8349
	1.5			1.9332	1.9999
	2.0			2.2716	2.3623
		0.2		15.9371	15.9999
		0.3		16.6546	16.7461
		0.4		17.0134	17.3240
			0.1	1.3450	1.5000
			0.4	1.3571	1.5032
			0.7	1.3634	1.4999

## 6. Conclusions

On the second-grade fluid flow produced by an exponentially expanding sheet, the consequences of activation energy and non-linear thermal radiation are examined. The following are some pertinent conclusions of the current analysis:

- The velocity sketch is increased by adding changes of the 2^nd^-order fluid parameter (*β*) and the magnetic field parameter (*M*).
- An increment in the estimations of parameter of induced magnetic field (*λ*) upsurges the profile of the induced magnetic field.
- By increasing in the variations of parameter of variable thermal conductivity (*ε*) as well as the Prandtl number (*P*_*r*_),hence, the temperature distribution has opposite behavior.
- For the stronger estimations of Schmidt number (*S*_*c*_), the magnetic field parameter (*M*) and Eckert number (*E*_*c*_) have the increasing behavior on the temperature distribution.
- By growing in the estimations of *S*_*c*_ and *δ*_*c*_, the concentration profile reduces.
- The growing in the estimations of *A*_*e*_ and *S*_1_ have the same behavior on concentration profile.
